# Mobile Health Interventions for Modifying Indigenous Maternal and Child–Health Related Behaviors: Systematic Review

**DOI:** 10.2196/57019

**Published:** 2025-04-30

**Authors:** Sana Ishaque, Ola Ela, Anna Dowling, Chris Rissel, Karla Canuto, Kerry Hall, Niranjan Bidargaddi, Annette Briley, Claire T Roberts, Billie Bonevski

**Affiliations:** 1 Flinders University College of Medicine and Public Health Flinders Health and Medical Research Institute Bedford Park Australia; 2 First Peoples Health Unit Griffith University Queensland Australia; 3 Lyell McEwin Hospital Northern Adelaide Local Health Network Elizabeth Australia

**Keywords:** Indigenous, co-design, mother, children, digital health, mobile health, mHealth, interventions, child health, maternal health, behavior, systematic review, effectiveness, lifestyle, postpartum, articles, literature, screening, PRISMA

## Abstract

**Background:**

Mobile health (mHealth) interventions promoting healthy lifestyle changes offer an adaptable and inexpensive method for accessing health information but require cultural appropriateness and suitability for acceptance and effectiveness in Indigenous populations. No systematic review on effective mHealth interventions for Indigenous women during pregnancy and the early childhood years has been conducted.

**Objective:**

This review evaluated the effectiveness of mHealth interventions promoting healthy behaviors for Indigenous mothers and children from conception to 5 years post partum. It also aimed to explore the observed effectiveness differences based on participant engagement, intervention design, and provision of context. Further, the review explored if the interventions were co-designed.

**Methods:**

A systematic search of 5 databases was conducted: SCOPUS, MEDLINE, CINAHL, PsycINFO, and ProQuest (Dissertation or Thesis). Studies were included if they were either a randomized controlled trial, pre-post comparison, or a cohort study using mHealth with Indigenous women for maternal and child health following a preregistered PROSPERO protocol (CRD42023395710). Health*Info*Net was searched for gray literature and the reference lists of included studies were hand searched. The initial title and abstract screen for eligibility were performed by 1 reviewer. A full-text screen of eligible studies and a quality appraisal of included studies was performed by 2 reviewers independently. The appraisal tools used were the Mixed Methods Quality Appraisal Tool and the Centre of Excellence in Aboriginal Chronic Disease Knowledge Translation and Exchange (CREATE). A descriptive synthesis of the extracted data was performed.

**Results:**

Of the 663 articles screened, only 3 met the eligibility criteria. Each paper evaluated a different mHealth intervention: Remote Prenatal Education; the SMS Parent Action Intervention (two-way text messaging); and the Screening, Brief Intervention and Referral to Treatment (SBIRT) eCHECKUP To Go (web-based screening and intervention). Statistically significant changes were reported in some outcomes, including an increase in the parental participation rate in face-to-face prenatal education; increased rate of breastfeeding initiation and exclusive breastfeeding (2-12 months); improved overall children’s behavior related to sleep, diet, physical activity, screen time, and intake of sugary beverages; improved individual children’s behavior related to physical activity and sleep; and decrease in alcohol drinks per week and binge drinking episodes per 2 weeks due to time effect. However, no study provided a sample size calculation for the reported significant outcomes. Also, due to the small number of included studies and each study evaluating a different intervention, it was not possible to combine results to ascertain if the participant engagement, intervention design, or community context had any impact on the effectiveness.

**Conclusions:**

Due to the lack of sample size calculation, it was not possible to establish whether differences in the effectiveness were due to the interventions or a type I statistical error. Therefore, caution is required in the interpretation of these findings.

**Trial Registration:**

PROSPERO CRD42023395710; https://www.crd.york.ac.uk/PROSPERO/view/CRD42023395710

## Introduction

Globally, Indigenous populations share experiences of devastating disruptions to their development due to violent settler colonial practices that persisted well into the twentieth century and continue to impact them today [1]. This historical suffering and the consequent vestiges are inextricably linked to the current overrepresentation of Indigenous people in population statistics relating to avoidable mortality, disease burden, and social and economic disadvantage [2]. To note, in this review, the term *Indigenous* is used for Indigenous populations across the globe.

Adverse health outcomes including low birth weight, preterm birth, stillbirths, and perinatal mortality rates disproportionately affect Indigenous families [3,4]. Indigenous women are more likely to experience domestic violence, have high levels of psychological distress, and exhibit a higher prevalence of behavioral risk factors including tobacco, alcohol, and other drug use in comparison with non-Indigenous women [5-7]. Disparities between Indigenous and non-Indigenous maternal and child health outcomes highlight the need for urgent and specialized support [3]. The perinatal period offers a unique opportunity for women to adopt healthy behaviors for the benefit of both themselves and their babies [8]. In addition, experiences during the first 2000 days of a child’s life have a significant, lasting impact on cognitive, physical, social, and emotional health [9,10]. Therefore, interventions applied during the perinatal period and early childhood are more successful than those implemented at a later stage of life [9].

Of relevance is the geographic impact on health inequities experienced by Indigenous populations. Compared with urban or regional populations, communities in rural and remote regions experience higher mortality [11]. The disparity is attributed to barriers associated with high cost, poor access, and culturally inappropriate service provision, as well as employment, education, and income disadvantages [12].

Mobile health (mHealth) interventions offer an alternative option for the provision and dissemination of health information, particularly for communities outside major cities where health services reach is reduced. Health service providers are increasingly using digital technology for health promotion due to its practicality, reach, and accessibility [13]. mHealth is being used for education, behavior modification, data collection and tracking, point-of-care diagnostics, health decision support, and record keeping [14,15]. This coincides with the increased use of mobile technology and specifically, its use for health and social and emotional well-being purposes by Indigenous women [16,17].

It has been established that Indigenous women are likely to successfully engage in services during the antenatal period when they feel empowered through education, family and community support, and cultural connectedness [4,6,18,19]. Effective health promotion is accessible, empowering, respects the values of Indigenous family structures and kinship systems, and most critically, is Indigenous-led [20]. Like any other intervention, mHealth when used for Indigenous populations requires the intervention to be culturally appropriate and acceptable [21].

There are very few studies that have reported the benefits of culturally responsive mHealth interventions specifically developed to support healthy lifestyle choices for Indigenous women [17,22,23]. Research that fails to consult with Indigenous communities has been shown to provide little benefit [10]. Applying homogenized, mainstream health interventions risks perpetuating inequities, hindering engagement, and discounting the legacy of historical atrocities [24]. It is critical that mHealth is culturally safe and acceptable and that evaluations consider differences in participant characteristics, features of the intervention, and the context of delivery [25].

The primary objective of this systematic review was to assess the effectiveness of mHealth interventions designed for use by Indigenous mothers (from conception to 5 years post partum) who seek to promote healthy behaviors. The secondary aims of this review included examining the observed differences in the effectiveness of the interventions based on participant engagement, the intervention design, and the programmatic and community context in which the intervention was implemented. This review also explored whether the mHealth intervention was designed or adapted specifically for Indigenous populations.

## Methods

A systematic review protocol outlining the search strategy, methods for title and abstract and full-text screening, data extraction template, quality assessment methods, data analysis methods, synthesis, statistical issues, publication bias, and any conflicts of interest was developed and registered with PROSPERO (CRD42023395710) [26]. The review followed the Preferred Reporting Items for Systematic Reviews and Meta-Analyses (PRISMA) guidelines for the conduct and reporting [27] (Multimedia Appendix 1). The protocol was followed fully except that the third aim, to explore if the identified interventions were co-designed, was included after the protocol was submitted for registration to PROSPERO based on experts’ advice.

### Eligibility Criteria

A publication was eligible for inclusion if it reported findings from a randomized controlled trial (RCT), pseudorandomized controlled trial, comparative study with concurrent controls, or pre-post study that applied a user-operated digital health intervention as an intervention. The population included was self-identified Indigenous women in the antenatal period or with children aged 0 to 5 years. The outcomes eligible for inclusion were health behaviors during pregnancy, which related to diet, use of tobacco, alcohol, other drugs, and physical activity; and behaviors performed by mothers or other caregivers during the early childhood period, including behaviors related to health and hygiene care, feeding, stimulation, responsiveness, and safety. The review was restricted to publications reported in English. There was no restriction on the type of control, type of digital health intervention, country, setting in which the study was conducted, or date of publication.

Studies were excluded if they reported on the development or design of a digital health intervention without reporting on its effectiveness on the behavioral outcomes of Indigenous women and children, reported on mixed Indigenous and non-Indigenous populations without reporting subgroup data, or applied the intervention directly to children with the intention to change maternal behavior.

In this review, user-operated digital health technologies are regarded as any form of computerized technology that the intended (beneficiary or target) audience interacts with in a way that is not mediated by a third party (eg, by a health care provider). This includes mobile phone technologies (or mHealth); web 2.0 technologies (inclusive of social media); websites and web-based applications; and non-web applications (eg, delivered via offline electronic devices).

### Search Strategy

With the assistance of a research librarian, a systematic search was performed in SCOPUS, MEDLINE, CINAHL, PsycINFO, and ProQuest (Dissertation or Thesis) in October 2022 and was updated in December 2023. The search was conducted using controlled vocabulary and keywords related to the terms *maternal*, *child and family* health, *Indigenous population*, and *mHealth*. Specially designed search filters have been used to identify terms for Indigenous populations [28]. Details of the search strategy for all databases are given in Multimedia Appendix 1. The search strategy was modified to adapt to variations in indexing among the other databases and a complete search strategy is available on request. In addition, the Health*Info*Net website and reference lists of the included studies were hand-searched for relevant gray literature and any additional relevant studies respectively.

### Relevance and Full-Text Screening

The literature screening was performed using the Covidence systematic review software. After removing duplicate articles, the title and the abstract screen were performed by 1 reviewer per article (SI, OE, and AD). The studies selected for full-text review were screened by 2 independent assessors (SI, OE, and AD) against the eligibility criteria, with conflicts managed with discussion and with the assistance of an expert reviewer (BB).

### Data Extraction and Analysis

Data extraction was performed by 1 assessor (either OE or AD) using a template designed to extract information about the study methodology, population characteristics (including self-identification of Indigenous status), behavioral target, intervention characteristics, intervention context, information about co-design of the intervention, and reported outcomes. Another reviewer (SI) checked the extracted data for accuracy and completeness.

The data are presented descriptively and in narrative form, where applicable. The planned analysis to perform meta-aggregation of qualitative data and meta-analysis of quantitative data was not feasible due to the small number of included studies.

### Risk of Bias Assessment

An assessment of the risk of bias for included studies was performed independently by two reviewers (OE, AD) using the Mixed Methods Appraisal Tool (MMAT) [29] and the Centre of Excellence in Aboriginal Chronic Disease Knowledge Translation and Exchange (CREATE) tool [30]; with a third assessor (SI) managing conflicts. Anticipating a relatively small body of literature, risk of bias assessment was not used to exclude otherwise eligible literature but is presented and discussed alongside the review results.

## Results

### Eligibility

**Figure 1:**
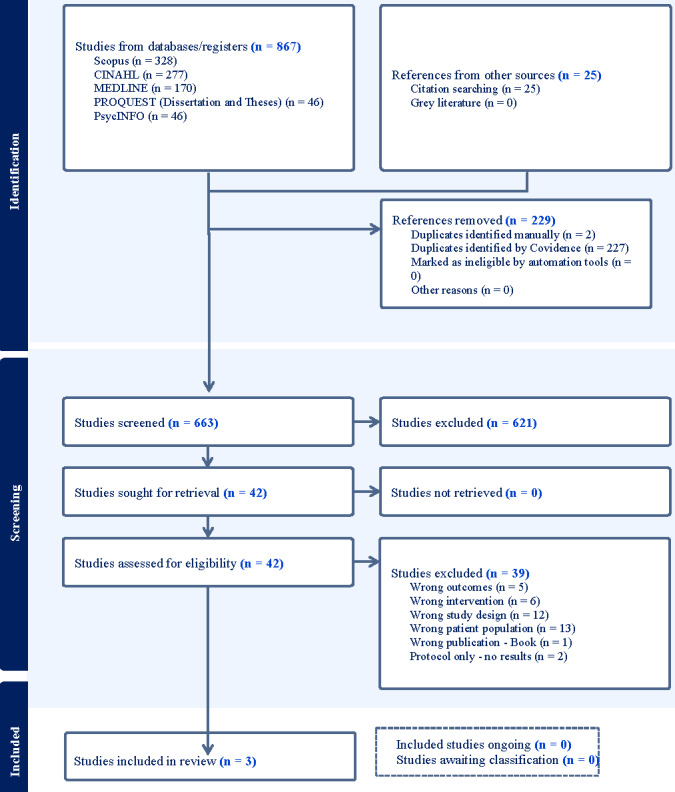
PRISMA (Preferred Reporting Items for Systematic Reviews and Meta-Analyses) flowchart.

From the database search, 867 articles were retrieved, of which 229 were duplicates. After the removal of duplicates, the title and the abstract of 663 articles were screened for relevance, of which 42 were found eligible for full-text screening. After the full-text screening of 42 articles, 3 met the inclusion criteria and were included in this systematic review (Figure 1) [31-33]. There were 39 excluded studies due to wrong outcome (5 studies), wrong intervention (6 studies), wrong study design (12 studies), wrong patient population (13 studies), wrong publication (ie, book; 1 study), and protocol only (ie, no results; 2 studies).

An additional 6 articles were found eligible for full-text screening after a hand search of reference lists of the included studies, but none of them were included in the review as the study designs were inappropriate for this review. Finally, 3 studies were included (Table 1).

The publication dates of the 3 included studies were 2021, 2019, and 2015 (Table 1) [31-33]. Two of the studies were conducted in the United States with American First Nation populations [31,33] and one was conducted in Canada [32] and included 3 First Nation communities of Canada (Sagkeeng, Sandy Bay, and Garden Hill). The study designs were pre-post comparison (n=2), and randomized controlled trial (n=1), respectively. The health conditions around which behavioral changes were sought included participation in the prenatal program and breastfeeding initiation [32], childhood obesity [31], and alcohol addiction (or problem drinking) [33]. The outcomes included breastfeeding initiation, breastfeeding duration, parental participation in parental education programs [31-33]; child behaviors related to sleep, diet, physical activity, screen time, and intake of sugary beverages [31]; and binge drinking and weekly drinking [33]. The interventions were applied in community settings in all 3 included studies.

**Table 1:** Study characteristics and outcomes.

Study ID	Study aims	Indigenous population and availability of internet	Country	Setting	Study design	Participant engagement	Participant characteristics and total participants (n)	Behavioral target	Outcomes reported
Hui et al [32]	To assess the impact of community-based remote prenatal education	Sagkeeng and Sandy Bay (two rural Ojibwe First Nation communities) Garden Hill (a remote Anishininew First Nation community) Internet available at all locations; Speed of Wi-Fi at Garden Hill unable to transfer high-quality video images	Canada	Community based	Pre-post comparison	42% attendance (n=53) in the communities for the social media-assisted prenatal chat group	Pregnant women Total number of participants not reported	Breastfeeding initiation Breastfeeding duration Parental participation in parental education program	Outcomes 1-year pre and post intervention: participation rate in prenatal programs across the 3 communities increased significantly; 36.4% (85/233 births) vs 54.5% (125/231 births; *P*<.001.); significant increase in breastfeeding initiation rate in only 1/3 of the communities: 23% (15/65) live births to 67% (288/42) live births (*P*<.001); no significant difference in breastfeeding initiation rate in Sandy Bay and Garden Hill areas.
Brown et al [31]	This study investigated the feasibility of delivering health-related short text messages to parents to reduce obesity risk among children aged 3 to 5 years	American Indian reservation	United States	Community based	Pre-post comparison	17/17 completed 5 weeks study	Parents of children 3 to 5 years American Indian (47%) Mean age 34 years, female (15/17, 88%) Annual incomes greater than US $30,000 (59%) College graduates (71%) Total n=17	Child behaviors related to sleep, diet, physical activity, screen time, and intake of sugary beverages	Outcomes compared pre- and postintervention: significant change in favor of the intervention in overall child behavior (*P*=.051); significant difference in child behavior related to physical activity and sleep (*P*=.046); no significant difference in child behavior related to diet
Montag et al [33]	To develop and test an adaptation of an online intervention for reducing risky drinking in Indigenous women of childbearing age	American Indian/Alaskan women	United States	Community based	Randomized controlled trial	263 recruited, 16 (6.1%) lost to follow up	Sample size for which demographics were reported n=247 Age: 28.6 ± 0.5 (28.6 ± 0.5) Intervention n=129, Control n=134, Total n=263	Binge drinking Weekly drinking	No significant difference between I and C for alcoholic drinks per week and binge episodes per 2 weeks. Alcoholic drinks per week and binge episodes per 2 weeks were significantly reduced due to the time effect *P*<.05.

### Study Interventions

The eHealth intervention Remote Prenatal Education in the pre-post study by Hui et al [32] was a substudy of a large intervention that also included some face-to-face components (Table 2). The eHealth component consisted of culturally tailored web-accessible educational material on topics related to the pregnancy, prenatal, and postnatal periods, such as healthy eating, recipes, traditional foods, food label reading, and nutrition content for common foods and a social media-assisted prenatal chat group operated through Facebook messenger. The chat was moderated by community elders, Canadian Prenatal Nutrition Program (CPNP) workers, or the study coordinator. The intervention was accessible via computer or smartphone and could be opened via Facebook. The educational material on the website was available as readings, audios, and videos; news on the prenatal or postnatal programming in the communities and traditional knowledge regarding a healthy pregnancy was also available. Traditional methods, including prayer, sharing circles, and storytelling, were incorporated in breastfeeding education. While the total number of participants was not reported, the participation rate for the social media prenatal group was 42% (n=53).

The pre-post study by Brown et al [31] investigated the Short Messaging System Parent Action Intervention. The study consisted of 3 short text messages per week for 5 consecutive weeks sent between 30 January and 17 March 2017 to 17 families (Table 2). The messages were tailored for the age and gender of participating parents and children and sent on the phone at individualized times of the day using an online text automation platform, Mosio. The text messages provided information about recommended healthy child nutrition, physical activity, sleep, and screen time behaviors of preschool children. The text message topics were derived from the Let’s Go! 5–2–1–0 US national campaign and recommendations for sleep in children.

The intervention in the randomized control trial used an online intervention called Screening, Brief Intervention, and Referral to Treatment eCHECKUP To Go; this screened participants for their use of alcohol, and based on their answers provided feedback about their risk of an alcohol-exposed pregnancy and its effect on the fetus (Table 2) [33]. After the initial screening, resources and information on support services were provided to participants. The intervention took approximately 20 minutes to complete. There were 129 women in the intervention group and 134 women in the control group that received “usual care,” which included nonspecific educational resources available in waiting areas.

**Table 2:** Intervention characteristics.

Study ID	Intervention name, details, whether it was live, and any links	Was the digital health intervention part of any bigger intervention (eg, face-to-face components)?	Intervention delivery and access method (eg, computer, tablet, phone)	Intervention context	Name of Indigenous nation or country	How many times did participants interact with the intervention (eg, text messages)?	Who delivered or moderated the intervention?
Hui et al 2021 [32]	Remote prenatal education via an educational website and social media–assisted prenatal chat groups (Facebook or Messenger)	Yes	Computer and smartphone (Facebook link with website)	Community-based remote and some face-to-face components	Sagkeeng and Sandy Bay (two rural Ojibwe Indigenous communities), Garden Hill (Anishininew Indigenous community)	Not reported	Community Elders, Canadian Prenatal Nutrition Program workers, and the study coordinator served as the hosts of the Facebook chat groups.
Brown et al 2019 [31]	Two-way text messaging: the Short Messaging System Parent Action Intervention. The SMS text message topics were derived from the Let’s Go! 5–2–1–0 US national campaign and recommendations	No	SMS text messages were sent using Mosio, an online platform for text message automation	Community	American and non-American Indians residing in American Indian Reserves	Three text messages each week for 5 consecutive weeks	Messages appeared to be sent from a local pediatrician or a tribal health dietitian
Montag et al 2015 [33]	Screening, Brief Intervention, and Referral to Treatment. eCHECKUP TO GO, a web-based brief assessment and intervention tool. Participants received individualized online feedback at the end of the session regarding their risk for an alcohol-exposed pregnancy, the impact of alcohol exposure to the fetus, the physical and financial cost of their alcohol consumption, and how their drinking compared with that of other Indigenous women. Approximately 20 minutes to complete.	No	Computer, phone, tablet (could be accessed by the website)	Participants were recruited from American Indian or Alaska Native health clinics (intervention delivered in community setting)	American Indian or Alaska Native women in Southern California	1, 3, and 6 months	Not applicable

### Study Outcomes

In the Hui et al [32] study, a significant increase was reported in the participation of pregnant women in a face-to-face prenatal program 1 year after the start of intervention compared with preprogram participation rates (Table 1). Furthermore, a significant increase was reported in the breastfeeding initiation rate at one of the study sites and exclusive breastfeeding between 2 to 12 months was also significantly increased at another study site.

In the Brown et al [31] study, a pre-post comparison of a 5-week text intervention, there was a significant increase in parent-reported overall child behaviors related to sleep, diet, physical activity, screen time, and intake of sugary beverages in favor of the intervention (Table 1). When individual behaviors were assessed, there was significant change in child behaviors related to physical activity and sleep, and there was no change in child behavior related to diet.

The Montag et al [33] study, which was a randomized controlled trial from April 2011 to September 2012, did not find any statistically significant differences in intake of alcoholic drinks per week and binge drinking episodes per week between the intervention and control groups (Table 1).

### Cultural Adeptness

The steps and strategies taken to ensure the cultural appropriateness of the mHealth interventions were extracted from the included studies (Table 3). Hui et al [32] and Montag et al [33] cited previous work that reported the interventions being co-designed for Indigenous populations, and Brown et al [31] used an intervention that was not reported to be co-designed or adapted for Indigenous populations. In the instances when previous research was cited, those articles were retrieved and relevant data were extracted (Table 3). Authors of the included studies were also contacted for information related to the co-design or adaptation.

**Table 3:** Cultural adaptation or community co-design approaches used in included interventions.

Study ID	Was the intervention culturally adapted (or co-designed)?	Were any citations provided about the co-design or adaptation of the intervention?	How was cultural adaptation done (eg, study design processes or co-design)?	What features made it culturally appropriate?	Were Indigenous people included in the design or adaptation?	Who else was included in the design or adaptation?	Theory for cultural adaptation or co-design	Evaluation of cultural appropriateness
Hui et al 2021 [32]	Yes	Yes [34]	Not reported	The intervention was built on the previous work of the researchers that identified barriers to participation among the study population [34]	Yes	None	Not reported	Not reported
Brown et al 2019 [31]	Not reported	No	Not reported	Research approved by the Institutional Review Board at the American Indian Tribal college on the reservation; the design, implementation, and interpretation were done in partnership	Yes	None	Not reported	Not reported
Montag et al2015 [33]	Yes	Yes [35]	Qualitative research and community outreach reported in authors’ previous work [35]	Inclusion of pictures and personal stories, emphasize confidentiality, incorporating family and community orientation, information tailored to the local community	Yes; Indigenous women clinical staff who had familiarity and experience with the topic and population	Non-Indigenous clinical staff who had familiarity and experience with the topic and population	Participatory research	Not reported

### Methodological Quality

The included studies were assessed for their methodological quality from an Indigenous perspective using the CREATE tool and MMAT (Tables 4 and 5 [36]; Multimedia Appendix 1). There was little information reported in the included articles that addressed the CREATE checklist. Authors of the included studies were contacted to obtain further information and, when provided (n=1), the information was used to appraise the studies. The following standards for measuring quality were applied based on the CREATE tool (Table 4); a study was considered high quality if 10 or more of 14 criteria were met, medium if 6-9 of 14 were met, and low if criteria 5 or below were met. Only Hui et al (2021) met the standards for high quality using this tool while the remaining 2 studies met the criteria for low-quality evidence [32]. The MMAT assessment (Table 5) returned a quality measurement of 43% (low-medium quality) for Hui et al [32], 57% (medium quality) for Brown et al [31], and 71% (medium quality) for Montag et al [33] [31-33]. This assessment was based on methods used in previous studies that define the quality as low, medium, or high according to the number of “yes” answers to questions [37,38]. If less than or equal to 25% of questions were in the affirmative, it was a low-quality study, medium if 50%, and high if >75% of questions were answered “yes” [30,37,38].

**Table 4:** Quality assessment with the Centre of Excellence in Aboriginal Chronic Disease Knowledge Translation and Exchange (CREATE) tool.

Study ID	Q1	Q2	Q3	Q4	Q5	Q6	Q7	Q8	Q9	Q10	Q11	Q12	Q13	Q14
Hui et al 2021 [32]	Y^a^	Y	Y	Y	Y	U^b^	U	Y	Y	Y	Y	Y	Y	Y
Brown et al 2019 [31]	P^c^	U	U	U	U	U	U	U	U	Y	U	Y	U	U
Montag et al 2015 [33]	Y	N^d^	U	N	N	N	N	N	U	N	U	Y	N	N

^a^Y: yes.

^b^U: unclear.

^c^P: partially.

^d^N: no.

**Table 5:** Quality assessment with the Mixed Methods Appraisal Tool.

Citation	Study design	SQ1	SQ2	Q1	Q2	Q3	Q4	Q5
Hui et al 2021 [32]	Cohort	Y^a^	Y	Y	N^b^	U^c^	U	N
Brown 2019 [31]	Before-after comparison	Y	Y	N	Y	N	Y	Y
Montag et al 2015 [33]	RCT	Y	Y	Y	Y	N	N	Y

^a^Y: yes.

^b^N: no.

^c^U: unclear.

## Discussion

This systematic review demonstrates that there is no evidence of the effectiveness of any currently available mHealth intervention designed for Indigenous women with young children (aged 0-5 years) to assist with behavior change for making healthy choices for themselves and their children. Only 3 articles found were eligible to be included in this systematic review. These studies aimed to change maternal and child behaviors in the prenatal period and preschool age group by provision of an mHealth intervention. The 3 mHealth interventions that were identified included a hybrid face-to-face and online prenatal education program; an automated, individualized, 3-times-per-week text message program giving healthy lifestyle advice for targeting preschool children; and a web-based self-assessment followed by tailored advice related to maternal alcohol drinking. All interventions were applied in a real-life community setting. A statistically significant change was reported in some behavioral outcomes. However, in the absence of any reported sample size or power calculation; it was not possible to ascertain if significant positive results were due to an inflated type I error, showing positive results in favor of intervention when in fact the interventions only worked due to very large sample sizes.

A recent review on barriers and facilitators of engagement of Indigenous peoples with mHealth interventions revealed themes that echo the need for effective and timely co-design practices in this field [39]. Researchers found that barriers to engagement included poor digital design, unreliable technology and internet, repetitive content with limited user input, language barriers, poor cultural representation, nonspecific health information, and privacy concerns [39]. Co-design has been identified as one of the most important factors determining the uptake of web-based therapeutic interventions for Indigenous populations [40]. In our review, it was deemed important to investigate if the digital interventions used were either co-designed in partnership with Indigenous communities or were adapted in consultation with the target population to ensure cultural appropriateness, or if they were selected from a pool of previous literature on culturally appropriate interventions for the target population. That information as reported in the included studies is presented in Table 3.

Of the 3 identified mHealth interventions, 2 were co-designed with an Indigenous population and showed some positive outcomes in favor of the interventions. Citations of their authors’ previous work on co-designing the interventions with Indigenous populations were reported for these 2 interventions in these studies [34,35]. Although the current evidence on their effectiveness is limited, the authors of these studies are following the iterative process of evidence gathering as suggested in the guideline on the evaluation of complex interventions [41-43]. Therefore, it will be important to see future results on the use of these interventions. Also, due to the diligent process of co-design and the assurance of cultural safety, these interventions may provide guidance on the planning and development of work with Indigenous populations on the provision of maternal behavior support. The consultation and partnership with communities reported by the 2 interventions are in line with the international guidelines on research with Indigenous peoples [44].

During the study selection process, 8 articles on the development of mHealth interventions for Indigenous women were identified that could not be included in this review [17,23,45-50]. Upon a further search with the names of the digital interventions reported in the studies, no follow-up studies on the evaluation of their effectiveness could be retrieved. Further to this, the corresponding authors of these articles (n=8) were contacted to inquire about any ongoing studies to evaluate their effectiveness. Except for 1, all authors replied to the initial email (7/8). Four authors (4/7) mentioned further evaluation work on the identified digital interventions, of which only 1 has already been published [51]. However, the focus of the publication was not an Indigenous population, therefore the study was not included in this review [51]. The other authors (n=3), with ongoing trials, were not able to share the unpublished data. Evidently, nearly 40% (3/8) of these mHealth interventions did not evaluate their effectiveness and are no longer active [17,23,48]. Although, in response to personal communication, one of the authors [23] mentioned the collection of qualitative data on the use of an app designed for Indigenous women [23], the need for an RCT on the evaluation of effectiveness was also identified. Consistent with the findings of our review, the evidence on the effectiveness of digital health interventions in other populations, such as children and adolescents [52], patients with heart failure [53], self-management of chronic obstructive pulmonary disease [54], and sexual health promotion [55] is limited due to the lack of published findings of well-designed and well-conducted effectiveness evaluation research. There are some health conditions for which the use of mHealth interventions has been well studied, such as asthma [56,57] and consumption of hazardous amounts of alcohol [58], in which the use of mHealth interventions has been shown to produce positive health outcomes in favor of the interventions [57,58]. Also, the use of mHealth to promote behavioral change during prenatal care in the general population has been reported to result in significant improvement in behavior risks, and improved healthy behaviors among pregnant women [59]. A current scoping review on the use and uptake of web-based therapeutic interventions among Indigenous populations across Australia, Canada, New Zealand, and the United States reported the use of these interventions for conditions such as cardiac care, diabetes, nutrition, mental health, asthma, neonatal care, otitis media, smoking cessation, and substance misuse [40]. For that review, a web-based therapeutic intervention was defined as a therapeutic intervention that was self guided, or a clinician-assisted program delivered via the internet to provide guidance, support, and treatment for health conditions. While the evaluation of effectiveness was beyond the scope of that review, it was concluded that these interventions have the potential to improve health, overcome treatment barriers, and reduce inequalities for Indigenous populations [40].

There are several factors that could lead to the lack of effective evaluation research and in turn scarcity of evidence on any mHealth interventions [60]. Some of these challenges pertain to interventions themselves, whereas some are related to designing a powered RCT with complex interventions, which is the common approach to effectiveness evaluation. Some of the challenges related to mHealth interventions are rapidly changing technology and the need for an intervention to evolve quickly to merely remain functional, let alone be upgraded; accessibility of the intervention across various operating systems and devices; acceptability and usability of the intervention by the target population; real-world use of any intervention or implementation; the burden on various stakeholders; adaptation of mHealth the intervention to context and population; and integration of an intervention into existing health care systems. There are also challenges specific to the design of an RCT for mHealth intervention evaluation, such as selecting a suitable context for trial so that the findings are generalizable, choice between conducting a trial with high external versus internal validity, specification, and explanation of the components of the mHealth intervention under evaluation, choice of an appropriate comparator group, and data collection methods from mHealth interventions. Given all these, it is arduous to conduct powered RCTs of mHealth interventions [41,60].

The included studies in this review were appraised for their quality with the CREATE and MMAT [36] tools. The CREATE tool was designed to ensure that the research conducted with Indigenous populations is conducted from the perspective of Indigenous peoples [30]. There are certain items that authors need to report for the assessors to mark the CREATE tool. While appraising the included studies in this review, we found that many of these items had not been reported and a further inquiry with the corresponding authors was necessary to access that information. This could have been due to the lack of any guidance on reporting of Indigenous research. Recently, a guideline was published on reporting of observational studies in Indigenous populations [61]; however, the studies included in this review were of different methodological design. Another reporting guideline, the Consolidated Criteria (CONSIDER) statement, developed with a review of available literature and a meta-synthesis, was published in 2019. The guideline provides 8 research domains and 17 criteria for the reporting of research involving Indigenous peoples. However, except for one, the included studies in the review were published before this date, so they cannot be discredited for not following the guideline.

Single data extraction was one limitation of this systematic review. Due to the paucity of available information, the data were extracted by 1 reviewer only. However, once extracted, the data were checked for completeness and errors by a second reviewer with more experience in conducting systematic reviews. This limitation is less likely to have changed the results and conclusions made, since the main concern is the lack of relevant literature and research in the field. Another limitation was the relatively small sample sizes in the included studies (n=420, n=17, and n=263), meaning the total number of women included in all studies was only 700. Furthermore, the lack of formal power calculations to calculate sample size and formally assess the statistical significance of the interventions was problematic. Also, the title and abstract screening for eligibility was performed by one reviewer. This can be perceived as a limitation as there is a risk of missing relevant studies, as they could be overlooked by a single reviewer. We, however, kept our decision-making more inclusive and the reviewer performing the title and abstract screening was instructed to include any study they were unsure about for the full-text screening stage. Another potential limitation may be the use of the CREATE tool for risk of bias assessment. The CREATE tool was developed with Indigenous and non-Indigenous experts and uses a 14-question checklist that assesses community engagement, consultation, research governance, and intellectual property to evaluate the methodology of studies conducted in the Australian Aboriginal and/or Torres Strait Islander population groups [30]. However, the tool has been widely used in research involving global Indigenous populations [62-67]. Therefore, the use of the CREATE tool provides additional information about the methodological suitability of the included studies from the Indigenous viewpoint. The addition of a third aim to the systematic review after the registration of the review protocol may be considered a potential weakness. This was, however, not included as a primary aim of the review and did not impact the conclusion made in the review. In addition, it was not possible to evaluate differences in the effectiveness of the identified interventions based on participant engagement, intervention nature, and context due to the small number of studies included. Also, each study used a different intervention and none of them reported power and sample size calculations. This may be perceived as a weakness of this systematic review. However, it is reflective of the nature of the research and literature available and demonstrates that more research is required in the field.

### Conclusion

Overall, the current evidence on the effectiveness of mHealth interventions used with Indigenous women is limited. However, there is potential for mHealth to effectively support Indigenous women with young children to improve their lifestyle choices for themselves and their children. The interventions that have been co-designed need to undergo further evaluation research for wider application. New interventions for different contexts and varied Indigenous nations are required to be developed, tested for evaluation, and deployed for population use.

## Appendix

Multimedia Appendix 1 PRISMA (Preferred Reporting Items for Systematic Reviews and Meta-Analyses) checklist.

## Abbreviations

CONSIDER: Consolidated Criteria
CPNP: Canadian Prenatal Nutrition Program
CREATE: the Centre of Excellence in Aboriginal Chronic Disease Knowledge Translation and Exchange
mHealth: mobile health
MMAT: Mixed Methods Appraisal Tool
PRISMA: Preferred Reporting Items for Systematic Reviews and Meta-Analyses
PROSPERO: International Prospective Register of Systematic Reviews
RCT: randomized controlled trial
